# Remembering Ronald Melzack (1929–2019)

**DOI:** 10.1080/24740527.2020.1757385

**Published:** 2020-06-01

**Authors:** Mary-Ellen Jeans, Joel Katz, Paul Taenzer

**Affiliations:** ME Jeans & Associates, Ottawa, ON, Canada; Department of Psychology, York University, Toronto, ON, Canada; Department of Physical Medicine and Rehabilitation, Queen’s University, Kingston, ON, Canada


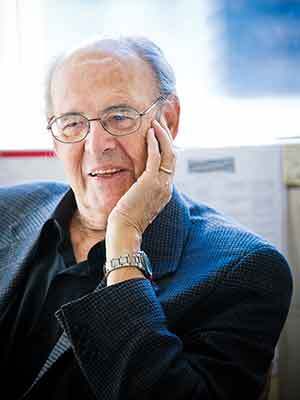
It is with great sadness that we acknowledge the passing of Professor Ronald Melzack on December 22, 2019. Our heartfelt condolences go to Ron’s beloved wife Lucy and their children Lauren and Joel. All of us in the international communities of pain research and clinical practice appreciate the enormous contributions Ron Melzack made to understanding the complexities of the neurophysiology and psychology of pain. These contributions continue to have a substantial impact on the burden of pain suffered by individuals and society.

Ron was born and raised in Montreal, Québec, Canada. His parents were immigrants to Canada, and he was their only child to attend university. He studied at McGill University where he earned a BSc, MSc, and, in 1954, a PhD in experimental psychology. His PhD studies and research were supervised by Professor Donald Olding Hebb, himself a world-renowned pioneer in brain circuitry underlying cognition. Ron’s PhD work led him to a long and illustrious career unraveling the neurophysiology of pain.

Following his PhD, Ron received several fellowships and postdoctoral positions from various prestigious institutions around the world. These included the University of Chicago; the University of Oregon; the Institute of Physiology, University of Pisa, Italy; University College, London, England; and the Massachusetts Institute of Technology (MIT), Boston. During his time at MIT, he met Professor Patrick Wall, a young physician and neuroanatomist from the UK. Together, they pondered the neural mechanisms underlying some of the more puzzling clinical phenomena—such as phantom limb pain and the absence of pain after traumatic injury—that were inconsistent with the predominant Cartesian model of pain. Melzack and Wall incorporated into a new theory, recent discoveries that descending pathways from the brain modulate activity in spinal cord dorsal horn interneurons. They proposed that sensory input from large- and small-diameter peripheral nerves was modulated by a gating mechanism in the spinal cord dorsal horns that was also influenced by descending signals from the cortex. The gate control theory, published in 1965 in the journal *Science*, provided a physiological explanation for how psychological phenomena such as attention, learning, past experiences, and emotions are incorporated into the experience of pain. It also provided an explanation for how traditional treatments, such as acupuncture, modulate pain, and it spawned novel treatments such as transcutaneous electrical nerve stimulation and spinal cord stimulation. The gate control theory fostered a “renaissance” in pain research and treatment. More than 50 years later, it is tempting to reflect on the global humanitarian impacts the new theory catalyzed. How many millions or perhaps billions of people have suffered less as a result of the basic science and clinical innovations that derived from Melzack and Wall’s insights into the nature of pain?

Prior to MIT, Ron spent several years as a postdoctoral fellow with William (Bill) Livingston at the University of Oregon, where he worked in the world’s first pain clinic interacting with patients in pain. This provided an opportunity for Ron to explore yet another vexing problem in clinical pain management and research: the lack of a valid tool for measuring pain. The origins of what was to become the McGill Pain Questionnaire (MPQ) began with Ron asking patients to describe what their pain felt like. Ron collected the words and, with a biostatistician colleague, Warren Torgerson, aggregated the pain descriptors into coherent groups based on pain quality and graded them by perceived intensity using interval scales. The MPQ provided a validated multidimensional measure of an individual’s pain experience and became widely used in both research and the clinic. Later, Ron would lead the development of a validated short form of the MPQ that has been translated into more than 20 languages and is still widely used in clinical trials. With the MPQ, Ron gave people in pain a way to describe the nuances of their experiences and provided clinicians and researchers with a tool to assess complex pain problems and measure the effectiveness of their interventions.

Ron returned to McGill University in 1963 where he assumed a full-time faculty position as associate professor in the Department of Psychology. He was later named the E.P. Taylor Professor of Pain Studies, a title he held from 1986 to 1999 when he was appointed Professor Emeritus.

Over the course of his career, Ron was actively involved with Canadian and international scientific and professional organizations. He was a fellow and member of the Canadian Psychological Association and was named honorary president from 1988 to 1989. In the 1970s, Ron, together with several U.S. and European colleagues, founded the International Association for the Study of Pain (IASP) and served as its president from 1984 to 1987 and member of its governing council from 1978 to 1990. The council met regularly in different countries around the world and, as part of their commitment to fostering advances in pain research and practice across the globe, offered lectures in their designated field in the host country. Ron was always a popular speaker, and he appealed to a diverse audience. During this stage of his career he furthered his influential role as an international leader in the field of pain, a reputation that lasted a lifetime.

Ron’s accomplishments continued. He collaborated with colleagues in the McGill community to develop Canada’s first multidisciplinary pain center. Together with the late Dr. Joseph Stratford, head of neurosurgery at the Montreal General Hospital (MGH), and Mary Ellen Jeans, who at the time was Ron’s first clinical psychology graduate student and a master’s prepared nurse, they founded the McGill/MGH pain clinic. From the outset, the clinic was meant to promote the application of new knowledge of pain, develop innovative approaches to pain management, and provide a much-needed service to patients suffering various types of chronic pain. It was specifically designed to be multidisciplinary, and several clinical specialties welcomed the service and voluntarily offered to assist, including psychology, nursing, physical therapy, psychiatry, and social work. Ron’s basic science PhD students and fellows often attended the pain clinic rounds, although they were not compelled to do so. This reflected Ron’s inclusive vision and his respect for all perspectives on pain. The pain clinic has evolved over the years and is now known as the Alan Edwards Pain Management Unit, an internationally recognized center of excellence for research and clinical practice. Ron would be proud of such a legacy.

In recognition of his ground-breaking accomplishments, Ron received many prestigious honors and awards. These included Officer of the Order of Canada, Prix du Québec, the Prix-Victorin, and investure as a Laureate in the Canada Medical Hall of Fame. He was appointed as a fellow of the Royal Society of Canada and as an officer of the Ordre national du Québec. He was awarded the Molson Prize for outstanding contributions to the arts, humanities, and social sciences presented by the Canada Council. Fittingly, Ron was presented the Donald O. Hebb Award for distinguished contributions to psychology as a science, the award for distinguished contributions to Pain Research and Management by the Canadian Pain Society, and the Killam Prize awarded by the Canada Council for the Arts. He also earned the Prix Adrien Pinard from the Société Québécoise pour la Recherche en Psychologie and the Canadian Psychological Association Gold Medal for distinguished and enduring lifetime contributions to Canadian psychology. Clearly Ron’s accomplishments were recognized by prestigious organizations nationally and internationally. An amazing record for an amazing man.

What is perhaps less known is Ron’s career-long interest in communicating with the lay public about the perplexing intricacies of pain and the need for pain to be clinical and research priorities. Ron published two articles in *Scientific American*, one in 1961 on pain perception and the second, in 1990, on the tragedy of needless pain. He also published two books for the lay public; *The Puzzle of Pain* (1973) and *The Challenge of Pain* (with Pat Wall, 2005). But what most people are surprised to discover is that Ron was the author of three children’s books in which he retells popular Inuit tales and legends. One of the stories, The Woman Who Raised a Bear as Her Son, from *The Day Tuk Became a Hunter*, was made into a feature cartoon film that won two awards in the early 1990s.

For those of us who had the privilege of working with Ron either as a colleague or as one of his doctoral or postdoctoral students, what we remember most are his humility, kindness, curiosity, enthusiasm, and persistence. Each of us has precious memories of how Ron inspired and supported us in our journeys. Ron’s legacy can be easily seen when we pause and reflect on the immense talent and collaborative spirit in the Canadian and international pain research and clinical communities.

It is not your responsibility to finish the work (of perfecting the world), but you are not free to desist from that either. (Rabbi Tarfon, Pirke Avot 2:16)

Some of you may recall this unusual passage from the dedication of Harold Merskey and Nikolai Bogduk’s seminal publication of the IASP’s Task Force on Taxonomy: *Classification of Chronic Pain*. Ron’s career epitomized Rabbi Tarfon’s teaching. Ron has surely done his part. Now that his work is done, we, as his intellectual offspring, are continuing to follow his example.

